# Inflammasome-Associated Gastric Tumorigenesis Is Independent of the NLRP3 Pattern Recognition Receptor

**DOI:** 10.3389/fonc.2022.830350

**Published:** 2022-03-01

**Authors:** Alice J. West, Virginie Deswaerte, Alison C. West, Linden J. Gearing, Patrick Tan, Brendan J. Jenkins

**Affiliations:** ^1^ Centre for Innate Immunity and Infectious Diseases, Hudson Institute of Medical Research, Clayton, VIC, Australia; ^2^ Department of Molecular Translational Science, Faculty of Medicine, Nursing and Health Sciences, Monash University, Clayton, VIC, Australia; ^3^ Cancer and Stem Cell Biology, Duke-NUS Graduate Medical School, Singapore, Singapore; ^4^ Genome Institute of Singapore, Singapore, Singapore; ^5^ Cancer Sciences Institute of Singapore, National University of Singapore, Institute of Singapore, Singapore, Singapore

**Keywords:** gastric cancer, inflammasomes, innate immunity, NLRP3, pattern recognition receptors

## Abstract

Inflammasomes are important multiprotein regulatory complexes of innate immunity and have recently emerged as playing divergent roles in numerous inflammation-associated cancers. Among these include gastric cancer (GC), the third leading cause of cancer-associated death worldwide, and we have previously discovered a pro-tumorigenic role for the key inflammasome adaptor apoptosis-associated speck-like protein containing a CARD (ASC) in the spontaneous genetic *gp130* ^F/F^ mouse model for GC. However, the identity of the specific pattern recognition receptors (PRRs) that activate tumor-promoting inflammasomes during GC is unknown. Here, we investigated the role of the best-characterized inflammasome-associated PRR, nucleotide-binding domain, and leucine-rich repeat containing receptor, pyrin domain-containing (NLRP) 3, in GC. In gastric tumors of *gp130* ^F/F^ mice, although NLRP3 expression was elevated at the mRNA (qPCR) and protein (immunohistochemistry) levels, genetic ablation of NLRP3 in *gp130* ^F/F^:*Nlrp3* ^-/-^ mice did not alleviate the development of gastric tumors. Similarly, cellular processes associated with tumorigenesis in the gastric mucosa, namely, proliferation, apoptosis, and inflammation, were comparable between *gp130* ^F/F^ and *gp130* ^F/F^:*Nlrp3* ^-/-^ mice. Furthermore, inflammasome activation levels, determined by immunoblotting and immunohistochemistry for cleaved Caspase-1, which along with ASC is another integral component of inflammasome complexes, were unchanged in *gp130* ^F/F^ and *gp130* ^F/F^:*Nlrp3* ^-/-^ gastric tumors. We also observed variable NLRP3 expression levels (mRNA and protein) among independent GC patient cohorts, and NLRP3 was not prognostic for survival outcomes. Taken together, these data suggest that NLRP3 does not play a major role in promoting inflammasome-driven gastric tumorigenesis, and thus pave the way for further investigations to uncover the key inflammasome-associated PRR implicated in GC.

## Introduction

Gastric cancer (GC) has the fifth highest incidence of cancer and is the third leading cause of cancer-associated death worldwide (1). The major histopathological type of human GC is intestinal-type adenocarcinoma, accounting for up to 90% of all cases, which develops stepwise from gastric inflammation (gastritis) to intestinal metaplasia, dysplasia, and ultimately adenocarcinoma (2, 3). Indeed, GC is among a growing number of cancers for which there is a link with chronic inflammation (at least 20%) triggered by dysregulated innate immune responses to microbial infection, in particular the *Helicobacter pylori* (*H. pylori*) bacterium that causes ~75% of all GC cases (4–9).

A role for innate immunity in GC was first suggested by the observation that polymorphisms in host genes that encode innate immune cytokines and/or their receptors, in particular *IL1B* encoding the pro-inflammatory cytokine interleukin-1 beta (IL-1β), conferred a marked increase in risk of *H. pylori*-infected individuals developing GC (10). Furthermore, transgenic over-expression of human IL-1β, or elevated endogenous expression of the IL-6 cytokine family member, IL-11, in mice can promote gastric tumorigenesis (11, 12). More recently, we and others have reported that polymorphisms and/or elevated expression of key regulators of the innate immune system belonging to the pattern recognition receptor (PRR) superfamily are associated with the onset and/or progression of GC, as well as poor survival outcomes (13–17).

PRRs are classified into several large structurally and functionally conserved subfamilies, among which include the well-documented Toll-like receptors (TLRs), absent in melanoma 2 (AIM2)-like receptors (ALRs), and nucleotide-binding oligomerization domain-containing (NOD)-like receptors (NLRs) (18–20). Collectively, PRRs play a critical role in coordinating host innate immune responses elicited against pathogenic microorganisms (e.g., *H. pylori*), as well as host-derived stress or damage signals (e.g., extracellular matrix components, heat shock proteins, and DNA from damaged or necrotic cells) (21, 22). In addition to their wide expression in immune cells of both innate (e.g., macrophages and neutrophils) and adaptive (e.g., B and T cells) arms of immunity, PRRs are also expressed in non-immune cells (e.g., epithelial, endothelial, and fibroblast). Indeed, with respect to this latter observation, it has emerged that PRRs can also directly elicit proliferation, survival, migration, and functional activation of these non-immune cells, which has relevance to numerous pathophysiological responses, including gut epithelial barrier integrity and oncogenic transformation (15, 20, 23). These diverse PRR-driven responses are transduced upon ligand engagement of PRRs *via* the activation of numerous signaling cascades, including nuclear factor kappa-light-chain-enhancer of activated B-cells (NF-κB), phosphatidylinositol 3-kinase (PI3K)/Akt, and mitogen activated protein kinases (MAPKs) (5, 22).

A subset of NLR family members (i.e., NLRC4, NLRP1, NLRP3, NLRP6, and NLRP12), as well as the AIM2 cytosolic DNA sensor, are best known for their assembly of large multiprotein complexes called inflammasomes, containing apoptosis-associated speck-like protein containing a CARD (ASC) and pro-Caspase-1, which control production of mature biologically active IL-1β and IL-18 cytokines (20, 24–26). The role of inflammasomes in cancer is complex, as evidenced by numerous studies coupling mice deficient in either ASC or Caspase-1 with various experimentally induced or spontaneous cancer models (e.g., colorectal cancer), which have revealed contrasting anti- or pro-tumorigenic functions for inflammasomes (26). This is evident also for inflammasome-associated PRRs, the best studied of which is NLRP3. For instance, *Nlrp3* ^-/-^ mice are more susceptible to chemical-induced colitis-associated colon cancer (CAC), whereas conversely, *Nlrp3* ^-/-^ mice are protected against tumor initiation and progression in a chemical-induced skin carcinogenesis model (27, 28). Although the mechanistic basis for the dual role of these ASC-containing inflammasome complexes remains to be fully elucidated, it is likely to be mediated by, at least in part, their differential expression and activity on distinct cell types (immune and non-immune), as well as usage of effector IL-1β and IL-18 cytokines.

In GC, we have recently reported a pro-tumorigenic role for ASC-containing inflammasomes in the *gp130* ^F/F^ mouse model for intestinal-type GC (29). These mice spontaneously develop inflamed gastric antral tumors at 6 weeks of age with 100% disease penetrance, which is caused by a homozygous phenylalanine (F) knock-in substitution of the cytoplasmic Y_757_ residue in glycoprotein (gp)130, the common signaling co-receptor for IL-6 family cytokines (30). Notably, genetic deficiency for ASC in *gp130* ^F/F^ mice suppressed gastric tumorigenesis by selectively downregulating the production of mature IL-18, but not IL-1β, which in the gastric tumor epithelium signals *via* NF-κB to protect GC cells against Caspase-8-like apoptosis (29). However, the identity of the inflammasome-associated PRR that promotes tumorigenesis in this model remains unknown. Here, we investigate the role of NLRP3 in inflammasome-associated gastric tumorigenesis and reveal that genetic ablation of NLRP3 in *gp130* ^F/F^ mice has no effect on early- or late-stage gastric tumorigenesis. In addition, *NLRP3* was differentially expressed among various GC patient cohorts, yet did not correlate with patient prognosis. Taken together, these data suggest that inflammasome-driven GC is independent of NLRP3.

## Materials and Methods

### Human Biopsies

Human gastric tissue biopsies were collected from GC or GC-free patients enrolled at Monash Medical Centre (Melbourne, Australia) undergoing surgical resection or upper gastrointestinal endoscopy (16, 17). Patients with a history of taking nonsteroidal anti-inflammatory drugs, proton pump inhibitors, or antibiotics were excluded. Biopsies were either snap-frozen in liquid nitrogen, or fixed in formalin prior to tissue embedding (paraffin) and sectioning on slides. Histopathological assessment and *H. pylori* status was determined on fresh GC patient tissue biopsies used for NLRP3 immunohistochemical staining (*n* = 6) as before (31). Full and informed patient consent was obtained, and biopsy collections were approved by the Monash Health Human Research Ethics Committee (13058A).

### Mice

The *gp130* ^F/F^ mice have been previously reported (30). Mice homozygous null for *Nlrp3* (32) were kindly provided by K. Fitzgerald (University of Massachusetts Chan Medical School, USA), and were used to generate *gp130* ^F/F^:*Nlrp3^-/-^* mice on a mixed 129Sv × C57BL/6 background. Mice were housed under specific pathogen-free conditions on a 12-h light/dark cycle, and all animal experiments were approved by the Monash University Monash Medical Centre “B” Animal Ethics Committee.

### Human GC Dataset Bioinformatic Interrogation

RNA sequencing (RNA-seq) gene counts for *NLRP3* and clinical data from The Cancer Genome Atlas (TCGA)-STAD cohort were obtained using the TCGAbiolinks R package. Counts were processed using the edgeR package. A DGEList object was created from the counts and gene annotation information was obtained using the Homo.sapiens package. Samples were grouped according to whether they were normal tissue (*n* = 32) or primary tumor (*n* = 375). Normalization factors were calculated using the TMM method and robust dispersions were estimated. Expression levels are expressed as log2 counts per million. The “Gastric Cancer Project ‘08 - Singapore Patient Cohort” (GSE15459) dataset was sourced for *NLRP3* gene expression profiling in tumor and non-tumor tissue (33). GC patient overall survival in both patient cohorts was assessed by Kaplan–Meier analysis on patients stratified into “low” versus “high” tumoral gene expression levels for *NLRP3* as described previously (13).

### Immunoblotting

Total protein lysates were prepared from snap-frozen mouse gastric tissues, and were subjected to immunoblotting and analysis as described before (17, 29). Antibodies used were those against mouse Caspase-1 (AdipoGen, AG-20B-0042-C100), mouse IL-18 (BioVision, 5180R-100), mouse IL-1β (R&D Systems, BAF401), mouse phospho(p)-NF-κB p65 (Ser536) (Cell Signaling Technology, 3031S), total NF-κB p65 (Cell Signaling Technology, 6956S), mouse ASC (AdipoGen, AG-25B-0006-C100), and α-tubulin (Abcam, Ab6160). Protein bands were visualized using the ECL method for Caspase-1 (p45/p20) or, for all other immunoblots, the Odyssey Infrared Imaging System (LI-COR). The bands were quantified using ImageJ software (34).

### Histology and Immunohistochemistry

Following formalin fixation and paraffin embedding (FFPE), histological assessment of mouse stomachs was performed blinded on 4- to 6-mm hematoxylin and eosin (H&E)-stained tissue sections. Immunohistochemistry on mouse gastric tissue sections was performed with primary antibodies against proliferating cell nuclear antigen (PCNA; Abcam, Ab18197), CD45 (550539) and B220 (550286) (BD BioSciences), CD3 (Abcam, Ab11089), cleaved Caspase-3 (9661S) and cleaved Caspase-8 (8592S) (Cell Signaling Technologies), and NLRP3 (R&D Systems, MAB7578), as described previously (17, 29). Immunohistochemistry on human gastric tissue sections was performed with a primary antibody against NLRP3 (R&D Systems, MAB7578). Positive-staining cells were counted manually on 50 counted cells per high-power field (HPF; *n* = 20) of 350 μm × 250 μm as described previously (17).

### RNA Isolation and Gene Expression Analyses

Snap-frozen mouse stomach tissues were subjected to mechanical homogenization on ice using a T 10 ULTRA-TURRAX^®^ instrument (IKA), following which total RNA was isolated using TRI Reagent^®^ Solution (Sigma), and subsequent on-column RNeasy^®^ Mini Kit RNA clean-up and DNase treatment (Qiagen). Total RNA was transcribed with the Transcriptor High Fidelity cDNA Synthesis Kit (Sigma-Aldrich), and quantitative real-time PCR (qPCR) was performed on cDNA with SYBR Green chemistry (Life Technologies) using the 7900HT Fast Real-Time System (Applied Biosystems), and the ViiA 7 Real-Time PCR System (ThermoFisher Scientific). Data acquisition and analyses were undertaken using the Applied Biosystems Sequence Detection System Version 2.4 software and ViiA 7 software. Sequences for mouse and human primers have been previously published (17, 29).

### Statistical Analyses

All statistical analyses were performed using GraphPad Prism V8.0.2 software. Statistical significance (*p* < 0.05) between the means of two groups was determined using Student’s *t*-tests (normal distribution) or Mann–Whitney *U* tests (abnormal distribution), and matched datasets involved Wilcoxon signed-rank tests. Statistical significance between the means of multiple groups was determined using ordinary one-way ANOVA (normal distribution) or Kruskal–Wallis (abnormal distribution) tests. All data are presented as the mean ± standard error of the mean (SEM) from at least 3 technical replicates. The log-rank test was used to calculate the statistical significance of the difference in survival between two groups. All data are presented as standard error of the mean (SEM).

## Results

### Differential Expression Status of *NLRP3* Among GC Patient Cohorts Does Not Correlate With Survival Outcomes

**Figure 1:**
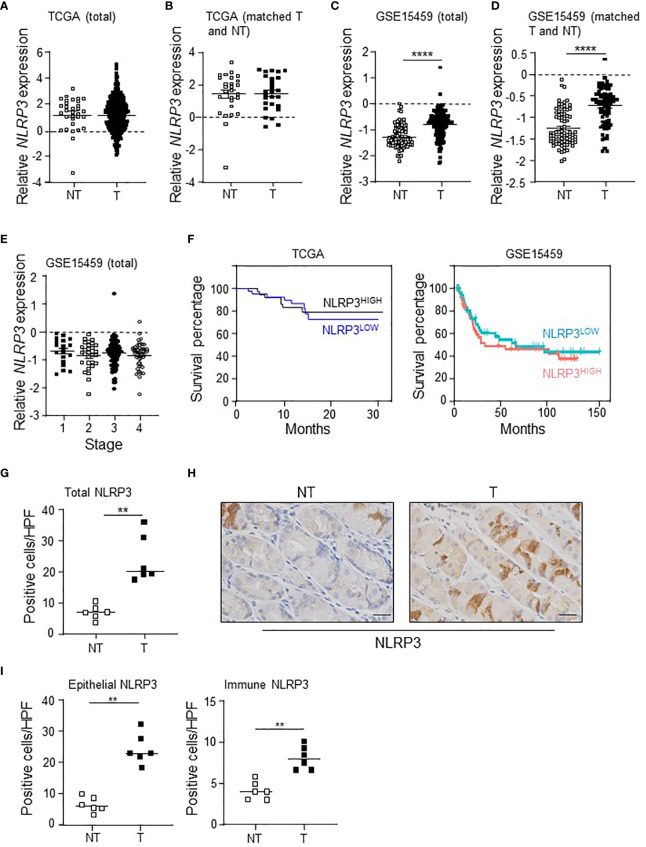
The differential expression of *NLRP3* in intestinal-type GC patient cohorts has no effect on survival outcomes. **(A, B**) *NLRP3* gene expression levels in **(A)** total gastric tumor (T; *n* = 375) and non-tumor (NT; *n* = 32) tissues, and **(B)** paired gastric tumor and adjacent non-tumor tissues (*n* = 27), from The Cancer Genome Atlas (TCGA) GC patient cohort. **(C, D)** *NLRP3* gene expression levels in **(C)** total gastric tumor (T; *n* = 177) and non-tumor (NT; *n* = 92) tissues, and **(D)** paired gastric tumor and adjacent non-tumor tissues (*n* = 83), from the “Gastric Cancer Project ‘08 - Singapore Patient Cohort” (GSE15459). In C, ****p < 0.0001, Mann–Whitney test. In D, ****p < 0.0001, Wilcoxon matched-pairs signed rank test. **(E)** Relative expression level of *NLRP3* among various disease stages [based on the American Joint Committee on Cancer TNM (tumor/lymph node/metastasis) 7th edition system] in the “Gastric Cancer Project ‘08 Singapore” Singaporean GC patient cohort (*n* = 177). Kruskal–Wallis test. **(F)** Kaplan–Meier survival analysis of the TCGA (*n* = 83) and GSE15459 (*n* = 138) GC patient cohorts stratified into 2 groups based on high and low *NLRP3* gene expression in tumors. **(G, I)** Quantification of NLRP3-positive immunohistochemical staining in **(G)** total cells or **(I)** epithelial versus immune cells, in the indicated groups of human gastric biopsies (*n* = 6 per sample group). **p < 0.01; Mann–Whitney test. **(H)** Representative photomicrographs (40×) of the indicated groups of human gastric biopsies that were immunostained with a human NLRP3 antibody. Scale bars: 20 μm.

Analysis of intestinal-type GC patient data within The Cancer Genome Atlas (TCGA) revealed that *NLRP3* mRNA levels were comparable between total gastric tumor tissues of patients (*n* = 375) compared to non-tumor gastric tissues (*n* = 32) (**Figure 1A**). In addition, *NLRP3* expression was similar in gastric tumor tissues compared to their paired adjacent non-tumor tissues (*n* = 27) (**Figure 1B**). Conversely, in a second independent intestinal-type GC patient cohort, the “Gastric Cancer Project ‘08 - Singapore Patient Cohort” (GSE15459) (33), comprising gastric tumor tissues (*n* = 177) and non-tumor gastric tissues (*n* = 92), *NLRP3* mRNA levels were significantly upregulated in total gastric tumor tissues (**Figure 1C**). Similarly, analysis of *NLRP3* expression in 83 patients for which there were paired tumor and adjacent non-tumor tissues indicated that *NLRP3* was significantly upregulated in tumors from 82% (68/83) of patients (**Figure 1D**). The altered tumoral expression of *NLRP3* did not align with a specific disease stage, since *NLRP3* mRNA expression levels were comparable in early (stage I and II) and advanced (stage III and IV) GC patients (**Figure 1E**). We also observed that in these independent GC patient cohorts, the segregation of GC patient primary tumors into either low or high *NLRP3* gene expression indicated that *NLRP3* mRNA levels are not prognostic for overall patient survival outcomes (**Figure 1F**). Furthermore, immunohistochemical staining on a third cohort (Australian) of GC patients (16, 17) indicated that NLRP3 protein expression levels were elevated in patient tumors compared to matched non-tumor gastric tissues, with increased numbers of diffusely stained NLRP3-positive cells observed throughout the transformed glandular epithelium and in immune cells in the stroma of patient tumor tissues (**Figures 1G–I**). Collectively, these divergent data suggest that NLRP3 may not have clinical significance in human GC.

### Genetic Targeting of NLRP3 in the *gp130* ^F/F^ GC Mouse Model Does Not Affect the Initiation or Progression of Tumorigenesis

**Figure 2:**
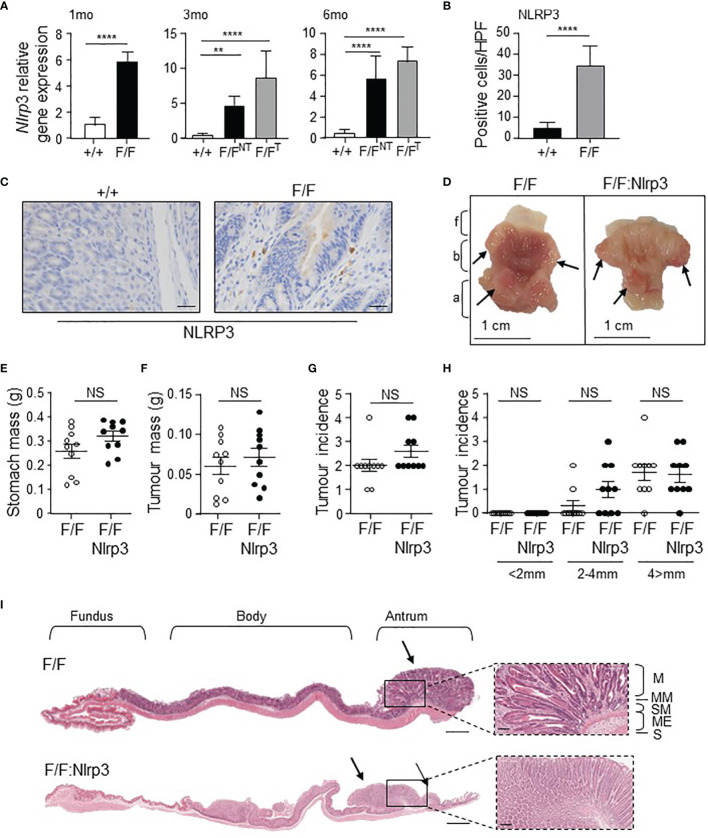
Genetic ablation of *Nlrp3* in 3-month-old *gp130* ^F/F^ mice does not suppress early gastric tumorigenesis. **(A)** qPCR expression analysis of *Nlrp3* in antral gastric tissue from 1-month-old (mo), 3mo, and 6mo wild-type (+/+) and *gp130* ^F/F^ (F/F) mice. NT, non-tumor. T, tumor. *n* = 6 per group. Expression data are normalized to *18S rRNA* and are presented from experimental triplicates as the mean ± SEM. **p < 0.01, ****p < 0.001; One-way ANOVA. **(B)** Quantitative enumeration of NLRP3-positive cells per high-power field (HPF) in gastric mucosa of wild-type and *gp130* ^F/F^ mice age 3 months (*n* = 6 mice per genotype). ****p < 0.001; Unpaired *t*-test. **(C)** Representative NLRP3-stained gastric antral cross-sections from 3mo wild-type and *gp130* ^F/F^ mice. Scale bars: 20 μm. **(D)** Representative 3mo *gp130* ^F/F^ and *gp130* ^F/F^:*Nlrp3* ^-/-^ (F/F:Nlrp3) mouse stomachs. Arrows indicate macroscopically visible tumors. Fundus (f), body (b), and antrum (a). **(E, F)** Total **(E)** stomach and **(F)** tumor mass of *gp130* ^F/F^ and *gp130* ^F/F^:*Nlrp3* ^-/-^ mice at 3 months of age (*n* = 10 per genotype). g, grams. NS, not significant; Unpaired *t*-test. **(G, H)** Graphs depict **(G)** total tumor incidence and **(H)** total tumor incidence by size (mm) in 3mo *gp130* ^F/F^ and *gp130* ^F/F^:*Nlrp3* ^-/-^ mouse stomachs (*n* = 10 per genotype). NS, not significant; Unpaired *t*-test. **(I)** Representative photomicrographs showing H&E-stained whole stomach sections from 3mo *gp130* ^F/F^ and *gp130* ^F/F^:*Nlrp3* ^-/-^ mice. Corresponding magnification (dotted inset) of the antral mucosa from the indicated genotypes. Scale bar: 1 mm (longitudinal sections) and 200 µm (magnification). Arrows point to macroscopically visible tumors. Right panel: M, mucosa; MM, muscularis mucosa; SM, submucosa; ME, muscularis external; S, serosa.

Since we have previously shown that elevated expression of the ASC inflammasome adaptor promotes gastric tumorigenesis in the spontaneous *gp130* ^F/F^ GC mouse model (29), we next assessed the expression of *Nlrp3* in these mice by qPCR. In this model, antral gastric intestinal-type tumors spontaneously develop from 1.5 months of age onwards with 100% penetrance, and progressively increase in size through to 6 months of age (13, 35). At 1, 3, and 6 months of age, gastric *Nlrp3* mRNA levels were significantly elevated (6- to 8-fold) in *gp130* ^F/F^ tumor-free gastric antrum (1 month old) or *gp130* ^F/F^ gastric antral tumors (3 and 6 months old) compared to either adjacent non-tumor antral tissue, or normal antral tissues of wild-type (WT) mice (**Figure 2A**). In support of these gene expression data, immunohistochemistry indicated that the total number of NLRP3 positively stained cells was significantly greater in *gp130* ^F/F^ antral tumors compared to normal antral tissue from WT mice, with predominant staining observed in the gastric tumor epithelium of *gp130* ^F/F^ mice (**Figures 2B, C**).

**Figure 3:**
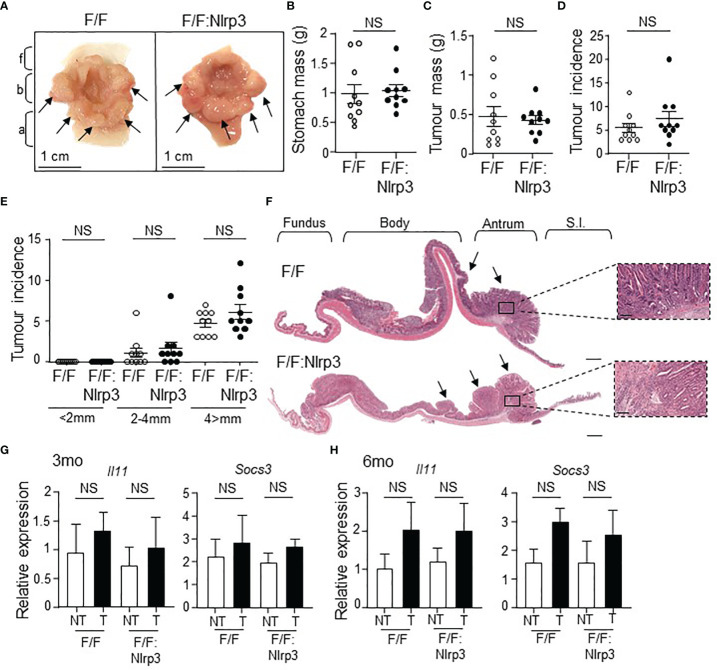
Genetic ablation of *Nlrp3* in 6-month-old *gp130* ^F/F^ mice does not suppress late-stage gastric tumor growth. **(A)** Representative 6-month-old (mo) *gp130* ^F/F^ and *gp130* ^F/F^:*Nlrp3* ^-/-^ (F/F:Nlrp3) mouse stomachs. Arrows indicate macroscopically visible tumors. Fundus (f), body (b), and antrum (a). **(B, C)** Total **(B)** stomach and **(C)** tumor mass of *gp130* ^F/F^ and *gp130* ^F/F^:*Nlrp3* ^-/-^ mice at 3 months of age (*n* = 10 per genotype). g, grams. NS, not significant; Unpaired *t*-test. **(D, E)** Graphs depict **(D)** total tumor incidence and **(E)** total tumor incidence by size (mm) in 6mo *gp130* ^F/F^ and *gp130* ^F/F^:*Nlrp3* ^-/-^ mouse stomachs (*n* = 10 per genotype). NS, not significant; Unpaired *t*-test. **(F)** Representative photomicrographs showing H&E-stained whole stomach sections from 6mo *gp130* ^F/F^ and *gp130* ^F/F^:*Nlrp3* ^-/-^ mice. Corresponding magnification (dotted inset) of the antral mucosa from the indicated genotypes. Scale bar: 1 mm (longitudinal sections) and 200 µm (magnification). Arrows point to macroscopically visible tumors. Right panel: M, mucosa; MM, muscularis mucosa; SM, submucosa; ME, muscularis external; S, serosa. **(G, H)** qPCR gene expression analysis of *Il11* and *Socs3* in antral gastric tissues from **(G)** 3mo and **(H)** 6mo *gp130* ^F/F^ and *gp130* ^F/F^:*Nlrp3* ^-/-^ mice. Expression data are normalized to *18S rRNA* and are presented from experimental triplicates as the mean ± SEM. NT, non-tumor; T, tumor. *n* = 6 per group. NS, not significant; Unpaired *t*-test.

To determine whether increased gastric tumoral expression of NLRP3 contributed to the pathogenesis of GC, we generated NLRP3-deficient *gp130* ^F/F^ mice (*gp130* ^F/F^:*Nlrp3* ^-/-^). At 3 months of age (early-stage tumorigenesis), the stomachs and gastric tumors of *gp130* ^F/F^:*Nlrp3* ^-/-^ mice were comparable in size and weight compared to age-matched *gp130* ^F/F^ littermate mice, as was the incidence of tumors (**Figures 2D–I**). Similarly, gastric tumorigenesis was also comparable in *gp130* ^F/F^:*Nlrp3* ^-/-^ and *gp130* ^F/F^ mice at 6 months of age (late-stage tumorigenesis), as evidenced by comparable stomach size and gastric tumor burden and incidence among age-matched mice of both genotypes (**Figures 3A–F**). Since tumorigenesis in *gp130* ^F/F^ mice is driven by IL-11-mediated hyper-activation of the STAT3 signaling pathway (11, 30), we also investigated whether the loss of NLRP3 affected this pro-tumorigenic signaling axis in the stomach. However, the expression of STAT3-target genes *Il11* and *Socs3* was similar among *gp130* ^F/F^:*Nlrp3* ^-/-^ and *gp130* ^F/F^ tumor and matched non-tumor gastric tissues at 3 and 6 months of age (**Figures 3G, H**). Therefore, these data indicate that NLRP3 does not contribute the onset and growth of gastric tumors in the *gp130* ^F/F^ GC model.

### NLRP3 Deficiency in *gp130* ^F/F^ Mice Does Not Reduce Gastric Inflammasome Activation

**Figure 4:**
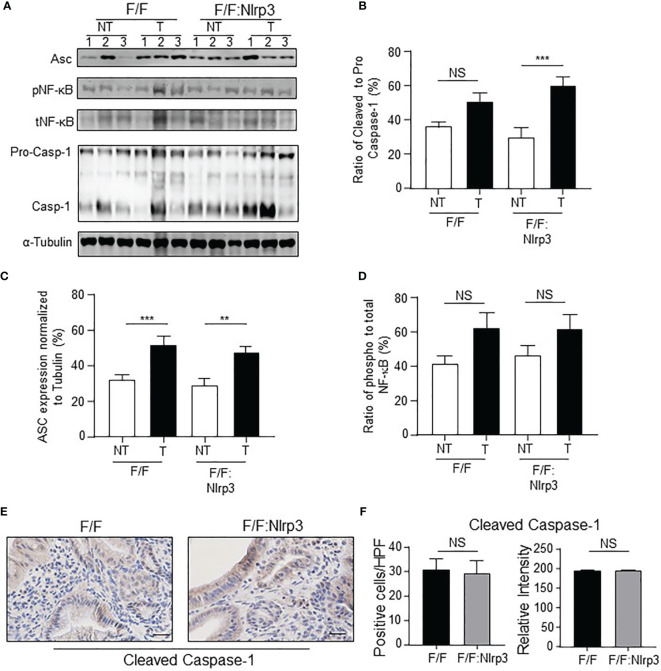
NLRP3 has no effect on inflammasome activation in the *gp130* ^F/F^ mouse model. **(A)** Immunoblots from 6mo *gp130* ^F/F^ and *gp130* ^F/F^:*Nlrp3* ^-/-^ gastric antral non-tumor and matched tumor tissue lysates with antibodies against ASC (22kDa), phospho (p) and total NF-κB (65 kDa), and Caspase-1 [both pro (45 kDa) and mature (20 kDa) forms are indicated]. Each lane represents an individual mouse. Protein loading was assessed using α-tubulin antibody. **(B–D)** Graphs depict densitometric quantification of immunoblots from individual antral and antral tumor tissue lysates (*n* = 6 mice per genotype) showing **(B)** ratio of cleaved to pro-Caspase-1, **(C)** ASC levels relative to α-tubulin, and **(D)** ratio of phospho to total NF-κB. **p < 0.01, ***p < 0.001; Unpaired *t*-test. NS, not significant. **(E)** Representative cleaved Caspase-1-stained gastric antral cross-sections from 6mo *gp130* ^F/F^ and *gp130* ^F/F^:*Nlrp3* ^-/-^ mice. Scale bars: 20 μm. **(F)** Quantitative enumeration of active Caspase-1-positive cells per high-power field (HPF) in gastric mucosa of 6mo *gp130* ^F/F^ mice and *gp130* ^F/F^: *Nlrp3* ^-/-^ mice (*n* = 6 mice per genotype). Unpaired *t*-test. NS, not significant.

Considering that NLRP3 activity is mediated *via* inflammasome complexes, and the gastric tumor phenotype in *gp130* ^F/F^ mice is associated with inflammasome activation (29), we investigated whether the ablation of NLRP3 affected inflammasome activation during gastric tumorigenesis. For this purpose, we compared the expression and/or activation status of key inflammasome components, ASC and Caspase-1, along with other inflammasome-associated PRRs, in gastric tissues from 6-month-old (mo) *gp130* ^F/F^:*Nlrp3* ^-/-^ and *gp130* ^F/F^ mice. Immunoblotting demonstrated that Caspase-1 activation levels, measured by detection of the cleavage of pro-Caspase*-*1 (p45) to its mature form (p20 subunit), were comparable between either gastric tumor or non-tumor tissues from *gp130* ^F/F^:*Nlrp3* ^-/-^ and *gp130* ^F/F^ mice (**Figures 4A, B**). Also, immunoblotting indicated comparable protein levels of ASC among *gp130* ^F/F^ versus *gp130* ^F/F^:*Nlrp3* ^-/-^ gastric tumor lysates (**Figures 4A, C**). Gene expression analyses by qPCR also indicated that NLRP3 deficiency did not significantly alter the mRNA levels of other inflammasome-associated PRRs in tumor samples from *gp130* ^F/F^ mice (**Supplementary Figure 1**). Furthermore, phosphorylation levels of NF-κB, which is activated downstream of inflammasomes to promote gastric tumorigenesis in the *gp130* ^F/F^ model (29), were also similar between *gp130* ^F/F^ and *gp130* ^F/F^:*Nlrp3* ^-/-^ gastric tumor or non-tumor samples (**Figures 4A, D**). Immunohistochemistry for cleaved Caspase-1 also confirmed comparable numbers and staining intensity for cleaved Caspase-1-positive cells throughout the tumor epithelium of *gp130* ^F/F^:*Nlrp3* ^-/-^ and *gp130* ^F/F^ mice (**Figures 4E, F**). Taken together, these findings further suggest that NLRP3 is not the primary inflammasome-associated PRR that contributes to gastric inflammasome activity and associated tumorigenesis in the *gp130* ^F/F^ model.

### NLRP3 Does Not Contribute to Gastric Tumor Cell Proliferation, Survival, or Inflammation During Tumorigenesis in the *gp130* ^F/F^ GC Model

**Figure 5:**
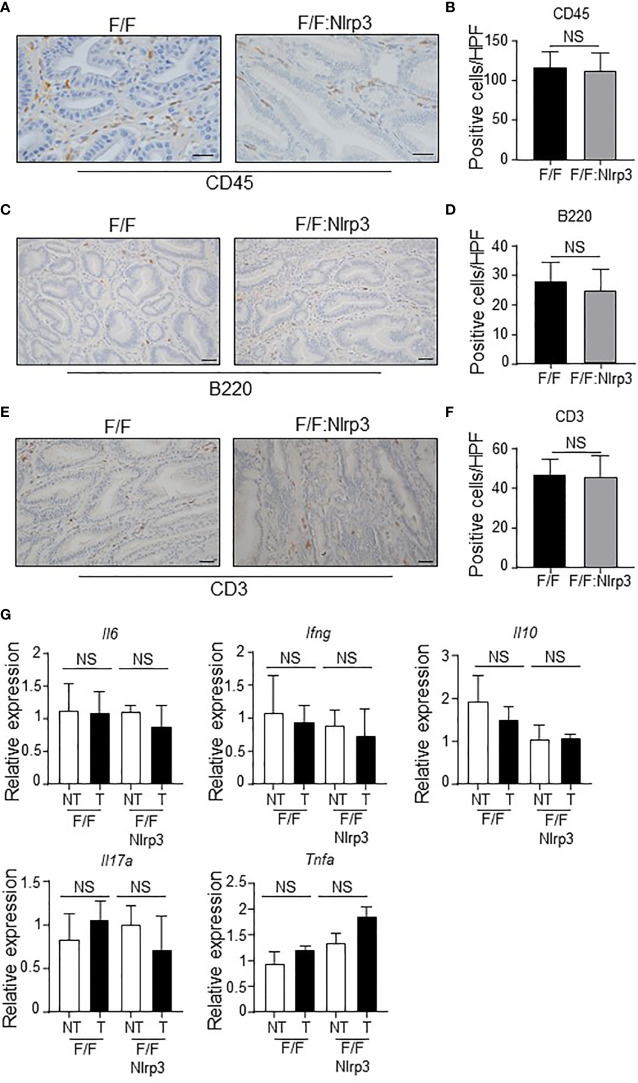
NLRP3 deficiency does not influence inflammatory responses during gastric tumorigenesis in the *gp130* ^F/F^ mouse model. **(A, C, E)** Representative **(A)** CD45-stained, **(C)** B220-stained, and **(E)** CD3-stained gastric antral tumor cross-sections from 6-month-old (mo) *gp130* ^F/F^ (F/F) and *gp130* ^F/F^:*Nlrp3* ^-/-^ (F/F:Nlrp3) mice. Scale bars: **(A)** 50 μm and **(C, E)** 20 μm. **(B, D, F)** Quantitative enumeration (mean ± SEM) of **(B)** CD45-positive, **(D)** B220-positive, and **(F)** CD3-positive cells per high-power field (HPF) in gastric tumor mucosa of the indicated 6mo mice (*n* = 6 mice per genotype). Unpaired t-test. NS, not significant. **(G)** qPCR expression analyses of inflammatory genes in gastric tumor and non-tumor tissues of 6mo *gp130* ^F/F^ and *gp130* ^F/F^:*Nlrp3* ^-/-^ mice (*n* = 6 mice/genotype). Expression data are normalized to *18S rRNA* and are presented from experimental triplicates as the mean ± SEM. Unpaired *t*-test. NS, not significant.

Despite NLRP3 ablation having no impact on gastric tumorigenesis in *gp130*F/F mice, it remained possible that NLRP3 may contribute to a subset of tumor-associated cellular processes in the gastric compartment. We first investigated whether NLRP3 deficiency in *gp130* ^F/F^ mice impacted on tumor-associated inflammation. However, immunohistochemistry analyses indicated comparable numbers of infiltrating CD45+ leukocytes, B220+ B cells, and CD3+ T cells in the gastric mucosa of 3mo and 6mo *gp130* ^F/F^ versus *gp130* ^F/F^:*Nlrp3* ^-/-^ mice (**Figures 5A–F** and **Supplementary Figures 2A–D**). The comparable gastric inflammation in *gp130* ^F/F^ and *gp130* ^F/F^:*Nlrp3* ^-/-^ mice was also confirmed at the molecular level, with qPCR analyses indicating similar mRNA levels of inflammatory genes (*Il6*, *Ifng*, *Il10, Il17a*, and *Tnfa*) among both genotypes at 3 and 6 months of age (**Figure 5G** and **Supplementary Figure 2E**).

**Figure 6:**
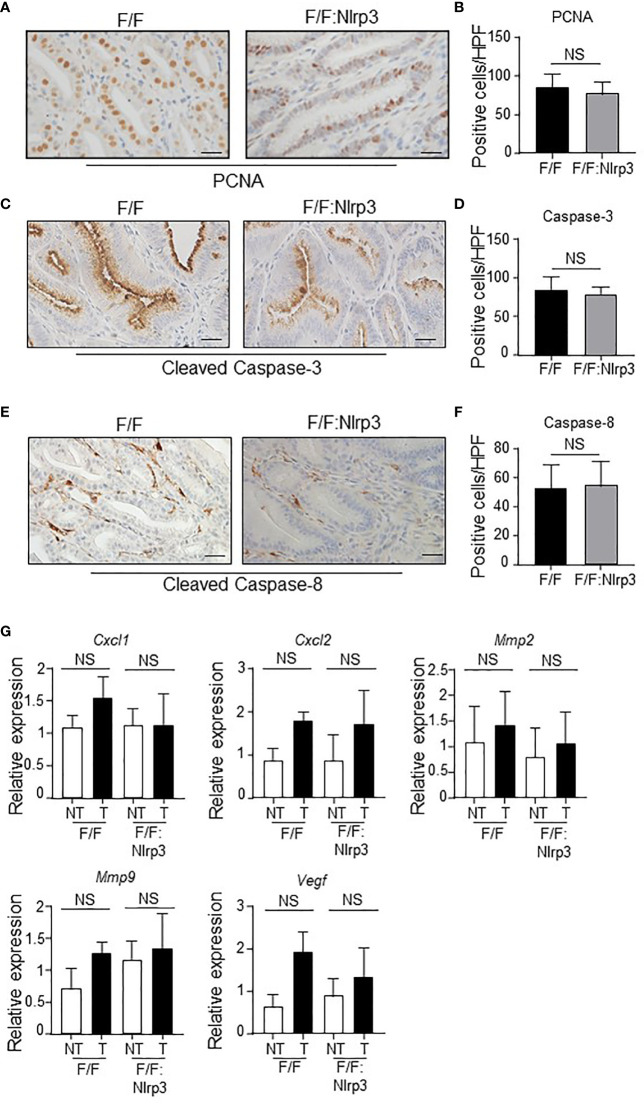
NLRP3 deficiency does not affect gastric tumor proliferation, and angiogenic and apoptosis markers in the *gp130* ^F/F^ mouse model. **(A, C, E)** Representative **(A)** PCNA-stained, **(C)** active Caspase-3-stained, and **(E)** active Caspase-8-stained gastric antral tumor cross-sections from 6-month-old (mo) *gp130* ^F/F^ (F/F) and *gp130* ^F/F^:*Nlrp3* ^-/-^ (F/F:Nlrp3) mice (*n* = 6 mice per genotype). Scale bars: 20 μm. (**B, D, F**) Quantitative enumeration (mean ± SEM) of **(B)** PCNA-positive, **(D)** active Caspase-3-positive, and **(F)** active Caspase-8-positive cells per high-power field (HPF) in gastric tumor mucosa of the indicated 6mo mice (*n* = 6 mice per genotype). Unpaired *t*-test. NS, not significant. **(G)** qPCR expression analyses of angiogenesis genes in gastric tumor and non-tumor tissues of 6mo *gp130* ^F/F^ and *gp130* ^F/F^:*Nlrp3* ^-/-^ mice (*n* = 6 mice/genotype). Expression data are normalized to *18S rRNA* and are presented from experimental triplicates as the mean ± SEM. Unpaired *t*-test. NS, not significant.

Since elevated gastric epithelial cell proliferation and survival are prominent features of inflammasome-associated gastric tumorigenesis in *gp130* ^F/F^ mice (29), we also investigated whether NLRP3 promoted these oncogenic cellular processes in the stomach. However, immunohistochemistry indicated similar PCNA^+^ proliferating cell numbers in the epithelium of gastric tumors of *gp130* ^F/F^ and *gp130* ^F/F^:*Nlrp3* ^-/-^ 3mo and 6mo mice (**Figure 6A, B** and **Supplementary Figures 3A, B**). Also, similar numbers of apoptotic cleaved Caspase-3^+^ and cleaved Caspase-8^+^ cells were observed in 3mo and 6mo *gp130* ^F/F^ and *gp130* ^F/F^:*Nlrp3* ^-/-^ mouse gastric tumors (**Figures 6C–F** and **Supplementary Figures 3C, D**). We also note that the expression of several angiogenesis-related genes (*Cxcl1, Cxcl2, Mmp2*, *Mmp9*, and *Vegf*) was comparable among gastric tumor and non-tumor tissues from 3mo and 6mo *gp130* ^F/F^ and *gp130* ^F/F^:*Nlrp3* ^-/-^ mice (**Figure 6G** and **Supplementary Figure 3E**). Collectively, these data suggest that NLRP3 does not contribute to key inflammasome-driven cellular processes that promote gastric tumorigenesis.

## Discussion

In cancer, it has recently emerged that inflammasome complexes exhibit contrasting pro- and anti-tumorigenic activities. This is evident for the best characterized NLRP3-containing inflammasome, whereby mice deficient in NLRP3 are more susceptible to azoxymethane/dextran sodium sulfate (AOM/DSS)-induced colitis-associated colon cancer (CAC) (27, 36). By contrast, comparable levels of tumor burden between wild-type and *Nlrp3* ^-/-^ mice when treated with AOM and DSS have also been observed (37). This dichotomy of function for NLRP3 in the intestine is also apparent in the chronic inflammatory setting of DSS-induced experimental colitis, in which *Nlrp3* ^-/-^ mice have been reportedly either more sensitive or resistant to intestinal inflammation and tissue damage (38, 39). While these findings highlight the complexity of NLRP3 functions in the intestine, interpreting these findings must be treated with caution since the DSS-induced colitis and AOM/DSS-induced CAC models are known for experimental discrepancies due to variable gut microbiota present in different animal housing facilities. Further highlighting the complex functions of NLRP3 in cancer is a study demonstrating that *Nlrp3* ^-/-^ mice are protected against experimentally induced inflammation-associated skin carcinogenesis (28).

To further explore the role of NLRP3 in cancer, our current study suggests that NLRP3 does not play a major role in promoting the pathogenesis of gastric inflammation-associated tumorigenesis. Using multiple independent GC patient cohorts, we observed variable levels of NLRP3 expression that did not align with survival outcomes, suggesting limited prognostic potential of NLRP3 in GC. Interestingly, polymorphisms in the *NLRP3* gene have been associated with increased GC risk in a Chinese patient cohort, and significantly upregulated NLRP3 expression levels in GC patient tumor biopsies have been correlated with poor prognosis, with progressively higher expression levels observed from gastritis to dysplasia and ultimately adenocarcinoma (40, 41). While our current study suggests otherwise, these contrasting disease associations for NLRP3 in GC, as is commonplace for many other PRRs in cancers, are most likely explained by the high genetic and molecular heterogenicity in human GC, together with differences in study design relating to patient characteristics (e.g., geographical location, ethnicity, age, tumor stage, histological grade, and anatomical location). We also acknowledge that in the context of the NLRP3 inflammasome, NLRP3 expression levels alone do not necessarily correlate with levels of NLRP3 inflammasome complex formation and activation. Therefore, a more thorough investigation of the expression and/or activation levels of NLRP3 inflammasome-associated components, namely, ASC, Caspase-1, and effector cytokines IL-1β and IL-18, is warranted in human GC patient samples.

Notably, our clinical findings were supported by data from the *gp130* ^F/F^ preclinical GC model, in which NLRP3 deficiency had no impact on tumor burden or well-documented tumor-promoting cellular processes, namely, cell proliferation and survival, inflammation, and angiogenesis. The *gp130* ^F/F^ mouse model was chosen for these studies since elevated inflammasome activation is a feature of ASC-driven gastric tumorigenesis in these mice (29). In addition, an advantage of *gp130* ^F/F^ mice compared to other GC mouse models (e.g., INS-GAS and Gan) is the short tumor latency of 6 weeks post-birth, coupled with 100% disease penetrance on multiple genetic backgrounds (129Sv, C57BL/6, and BALB/c) (11, 30).

Although the role of NLRP3 in GC has been poorly investigated to date, it is worth comparing our findings with a recent study using human cell lines, in which it was suggested that the NLRP3 inflammasome (*via* IL-1β) promoted GC cell proliferation and tumorigenesis by upregulating transcription of the cell cycle regulatory gene, *CCND1* (41). However, one must caution the interpretation of these experimental findings since this study involved the use of mis-identified or cross-contaminated human GC cell lines (e.g., BGC-823 and SGC-7901) (42, 43). Since our current *in vivo* data are generated from the *gp130* ^F/F^ model for intestinal-type GC, which is the predominant subtype of GC, our findings now warrant further investigations on NLRP3 in additional genetically defined models for GC, including those for the diffuse type of GC.

At present, the role of PRRs in driving the pathogenesis of GC is not well defined. Current evidence from preclinical models suggests that TLR2 and the AIM2 cytosolic DNA sensor drive gastric tumorigenesis, independent of inflammation, by cancer cell autonomous mechanisms that augment the proliferation and survival (TLR2), as well as migration (AIM2), of the gastric tumor epithelium (13, 17). Of note, AIM2 was shown to promote gastric tumorigenesis independent of inflammasomes *in vivo*, including in the *gp130* ^F/F^ mouse model (17). Since gastric tumorigenesis in the *gp130* ^F/F^ GC model is driven in part by ASC-containing inflammasomes, this genetic model provides an invaluable tool to dissect the contribution of distinct inflammasome-associated PRRs to GC. Since the global knockout of NLRP3 in *gp130* ^F/F^ mice did not alter inflammasome activation, NLRP3 does not appear to be the primary inflammasome-activating PRR that is associated with gastric tumorigenesis. Our study therefore paves the way to further investigate the identity of the inflammasome-associated PRRs, for instance, other members of the NLR family (e.g., NLRP1 and NLRC4), that contribute to the pathogenesis of GC.

## Data Availability Statement

The raw data supporting the conclusions of this article will be made available by the authors, without undue reservation.

## Ethics Statement

The studies involving human participants were reviewed and approved by Monash Health Human Research Ethics Committee. The patients/participants provided their written informed consent to participate in this study. The animal study was reviewed and approved by Monash University MMC “B” Animal Ethics Committee.

## Author Contributions

Study conception and design: BJJ. Acquisition of study data: AlicW, VD, AlisW, LG, PT, and BJJ. Analysis and interpretation of data: AW, VD, LG, and BJJ. Writing and/or revision of the manuscript: AlicW and BJJ. Study supervision: BJ. All authors contributed to the article and approved the submitted version.

## Funding

This work was funded (ID 1139371) by the National Health and Medical Research Council (NHMRC) of Australia (BJJ) and the Operational Infrastructure Support Program by the Victorian Government of Australia. AlicW was supported by an Australian Postgraduate Award from the Australian Government, as well as the Daniel Wilson-Metafit Australia Postgraduate Research Scholarship. BJ was supported by an NHMRC Senior Medical Research Fellowship.

## Conflict of Interest

The authors declare that the research was conducted in the absence of any commercial or financial relationships that could be construed as a potential conflict of interest.

## Publisher’s Note

All claims expressed in this article are solely those of the authors and do not necessarily represent those of their affiliated organizations, or those of the publisher, the editors and the reviewers. Any product that may be evaluated in this article, or claim that may be made by its manufacturer, is not guaranteed or endorsed by the publisher.
