# Supplementary material for: Inflammasome-Associated Gastric Tumorigenesis Is Independent of the NLRP3 Pattern Recognition Receptor
Source: Front Oncol. 2022 Mar 1;12:830350. doi: 10.3389/fonc.2022.830350 (PMC8921257; doi:10.3389/fonc.2022.830350)
Supplement: Supplementary file 1 [file DataSheet_1.pdf]

Supplemental Information

Inflammasome-associated gastric tumourigenesis is independent of the NLRP3 pattern recognition receptor

Alice J. West, Virginie Deswaerte, Alison C. West, Linden J. Gearing, Patrick Tan, and Brendan J. Jenkins.

Inventory of Supplemental Data

Supplementary Figure S1 Gene expression analysis of other inflammasome-associated pattern recognition receptors in 6-month-old *gp130*$^{F/F}$ and *gp130*$^{F/F}$:*Nlrp3*$^{-/-}$ mice.

Supplementary Figure S2 NLRP3 deficiency does not influence inflammatory responses during early gastric tumourigenesis in 3-month-old *gp130*$^{F/F}$ mice.

Supplementary Figure S3 NLRP3 deficiency does not affect gastric tumour proliferation, angiogenic and apoptosis markers in 3-month-old *gp130*$^{F/F}$ mice.

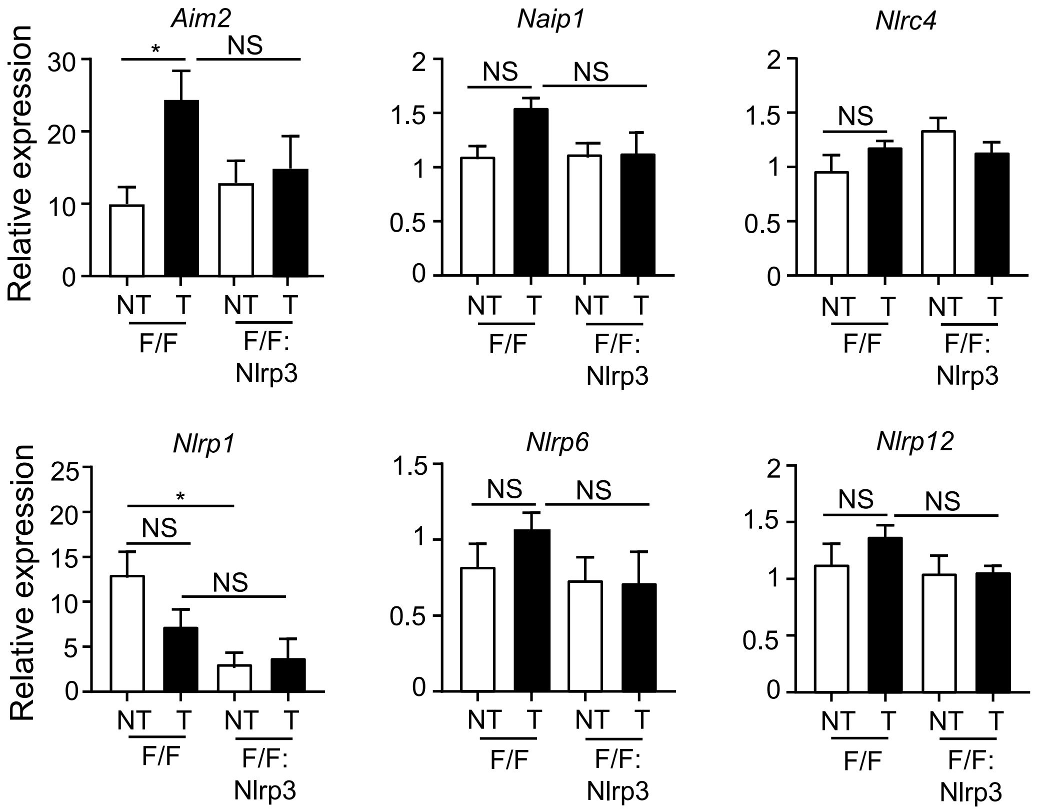

Supplementary Figure S1

Gene expression analysis of other inflammasome-associated pattern recognition receptors in 6-month-old *gp130*$^{F/F}$ and *gp130*$^{F/F}$:*Nlrp3*$^{-/-}$ mice. qPCR expression analyses of inflammasome-associated pattern recognition receptor genes in gastric tumour (T) and non-tumour (NT) tissues of 6mo *gp130*$^{F/F}$ and *gp130*$^{F/F}$:*Nlrp3*$^{-/-}$ mice (n = 6 mice per genotype). Expression data are normalized to *18S rRNA* and are presented from experimental triplicates as the mean ± SEM. **P* < 0.05; One-way ANOVA. NS, not significant.

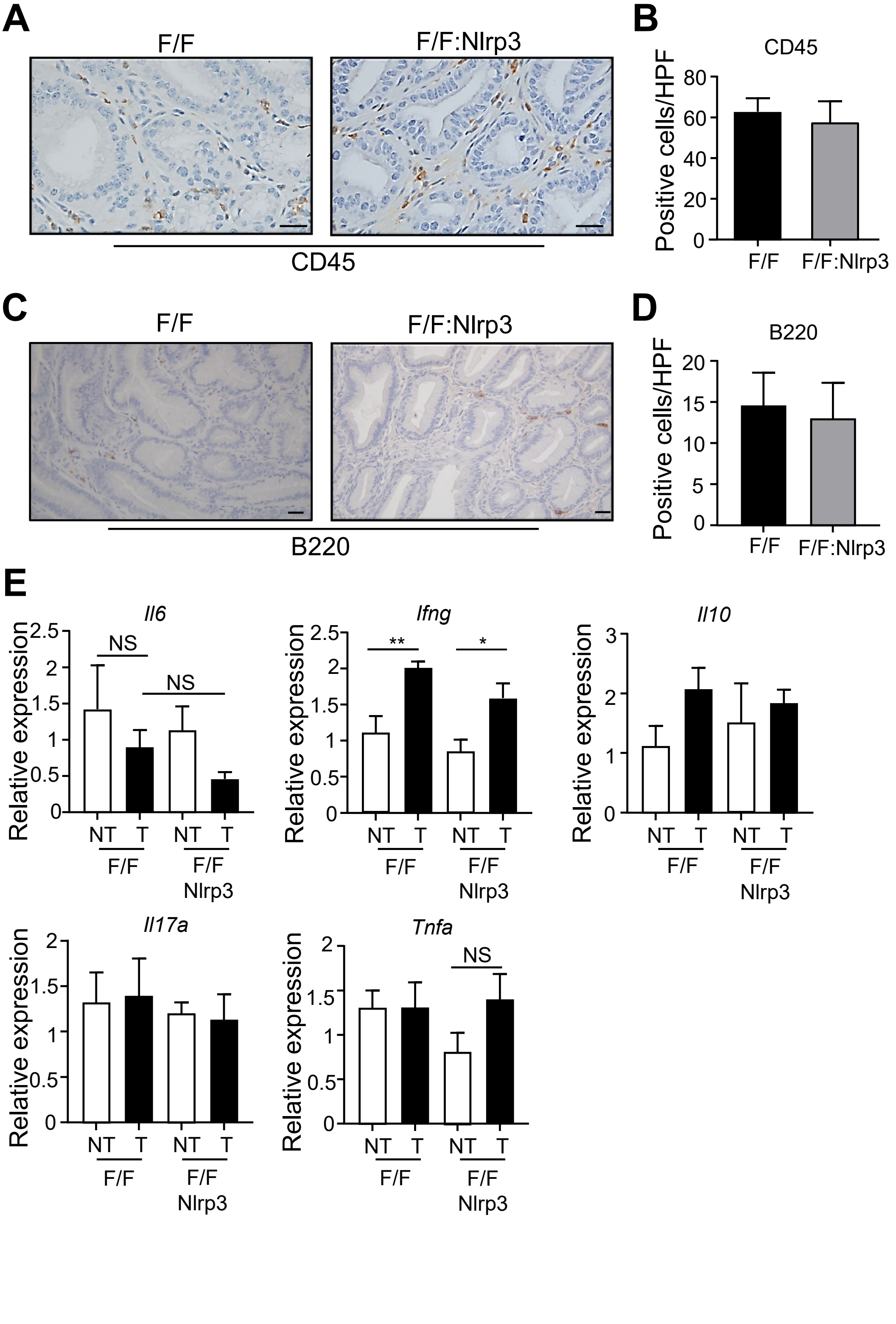

Supplementary Figure S2

NLRP3 deficiency does not influence inflammatory responses during early gastric tumourigenesis in 3-month-old *gp130*$^{F/F}$ mice. **A** and **C**, Representative (A) CD45- and (C)

B220-stained gastric antral tumour cross-sections from 3-month-old (mo) *gp130*$^{F/F}$ (F/F) and *gp130*$^{F/F}$:*Nlrp3*$^{-/-}$ (F/F:Nlrp3) mice. Scale bars, 100μm. **B** and **D,** Quantitative enumeration (mean ± SEM) of (B) CD45- and (D) B220-positive cells per high-power field (HPF) in gastric tumour mucosa of the indicated 3mo mice (n = 6 mice per genotype). **E,** qPCR expression analyses of inflammatory genes in gastric tumour and non-tumour tissues of 3mo *gp130*$^{F/F}$ and *gp130*$^{F/F}$:*Nlrp3*$^{-/-}$ mice (n = 6 mice per genotype). Expression data are normalized to *18S rRNA* and are presented from experimental triplicates as the mean ± SEM. *$P < 0.05$, **$P < 0.01$; One-way ANOVA. NS, not significant.

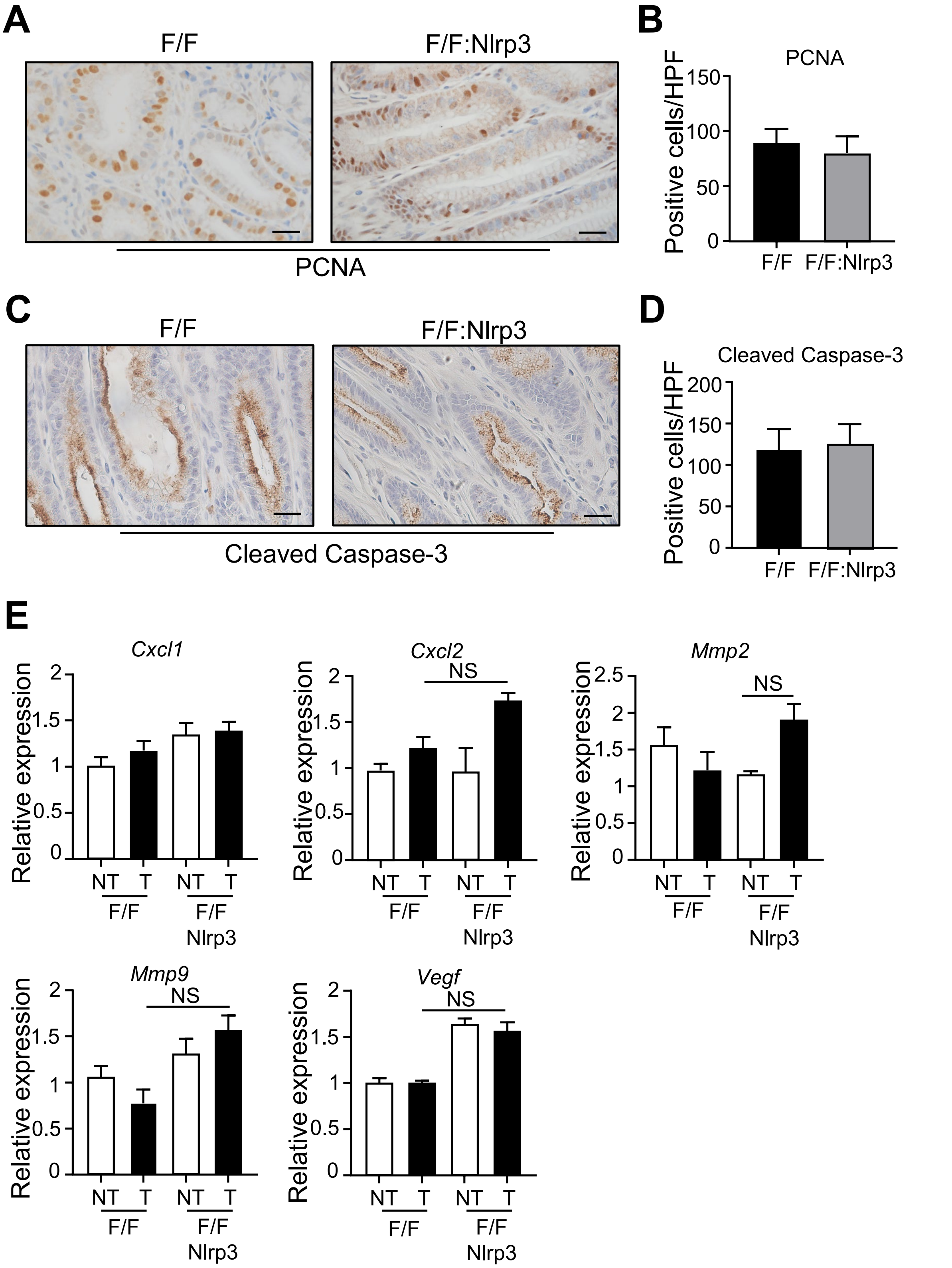

Supplementary Figure S3

NLRP3 deficiency does not affect gastric tumour proliferation, angiogenic and apoptosis markers in 3-month-old *gp130*$^{F/F}$ mice. **A and C** Representative (A) PCNA- and (C) active

Caspase-3-stained gastric antral tumour cross-sections from 3-month-old (mo) *gp130*$^{F/F}$ (F/F) and *gp130*$^{F/F}$:*Nlrp3*$^{-/-}$ (F/F:Nlrp3) mice (n = 6 mice per genotype). Scale bars, 100μm. **B** and **D,** Quantitative enumeration (mean ± SEM) of (B) PCNA- and (D) active Caspase-3-positive cells per high-power field (HPF) in gastric tumour mucosa of the indicated 6mo mice (n = 6 mice per genotype). **E,** qPCR expression analyses of angiogenesis genes in gastric tumour and non-tumour tissues of 3mo *gp130*$^{F/F}$ and *gp130*$^{F/F}$:*Nlrp3*$^{-/-}$ mice (n = 6 mice/genotype). Expression data are normalized to *18S rRNA* and are presented from experimental triplicates as the mean ± SEM. One-way ANOVA. NS, not significant.
